# Anti‐neuroinflammation effects of transcutaneous auricular vagus nerve stimulation against depression‐like behaviors via hypothalamic α7nAchR/JAK2/STAT3/NF‐κB pathway in rats exposed to chronic unpredictable mild stress

**DOI:** 10.1111/cns.14207

**Published:** 2023-04-10

**Authors:** Yu Chen, Yue Zhang, Junying Wang, Shaoyuan Li, Yifei Wang, Zixuan Zhang, Jinling Zhang, Chen Xin, Yu Wang, Peijing Rong

**Affiliations:** ^1^ Department of Physiology, Institute of Acupuncture and Moxibustion China Academy of Chinese Medical Sciences Beijing 100700 China; ^2^ Institute of Basic Research in Clinical Medicine China Academy of Chinese Medical Sciences Beijing 100700 China

**Keywords:** depression, neuroinflammation, transcutaneous auricular vagus nerve stimulation, α7 nicotinic acetylcholine receptor

## Abstract

**Background:**

Transcutaneous auricular vagus nerve stimulation (taVNS) is a vital neuromodulation for the treatment of depression, but its antidepressant molecular mechanism is unclear. The α7 nicotinic acetylcholine receptor (α7nAchR) is a key mediator of the vagus nerve that mediates its anti‐inflammatory efficacy. Here, we investigated whether the antidepressant effect of taVNS in chronic unpredicted mild stress (CUMS)‐exposed rats works through the α7nAchR/JAK2/STAT3/NF‐κB pathway.

**Methods:**

The depression model was established by CUMS for continuous 6 weeks in rats. From the 4th week of the experiment, CUMS‐exposed rats were subjected to taVNS for 3 weeks. To clarify the role of α7nAchR in the antidepressant effect of taVNS, we used α7nAchR^−/−^ gene knockout rats. The sucrose preference test (SPT), open field test (OFT), and forced swimming test (FST) were used to evaluate depression‐like behaviors of rats. Immunofluorescent staining was used to observe the morphology of microglia in the hypothalamus. Western blot was used to examine the protein expression of α7nAchR, p‐JAK2, p‐STAT3, IL‐1β, NF‐κB p65, and p‐NF‐κB p65 in the hypothalamus.

**Results:**

Depression‐like behaviors in CUMS‐exposed rats were manifested by decreased SPT ratio, increased FST immobility time, decreased total distance, vertical movement score, and activity time of OFT. Hypothalamic neuroinflammation in CUMS‐exposed rats was manifested by an amoebic‐like activated state of microglia, downregulated expression of α7nAchR, p‐JAK2, p‐STAT3, and upregulated expression of NF‐κB p65, p‐NF‐κB p65, and IL‐1β. TaVNS could significantly reverse the above‐mentioned phenomena, but had a poor improvement effect for CUMS‐exposed α7nAchR^−/−^ rats.

**Conclusion:**

The hypothalamic α7nAchR/JAK2/STAT3/NF‐κB signaling pathway may play an important role in the antidepressant‐like behavior of taVNS.

## Graphical abstract

Transcutaneous auricular vagus nerve stimulation (taVNS) activates the nucleus of tract solitary by stimulating the auricular branch of the vagus nerve and then projects to the hypothalamus, which inhibits the activated microglia. TaVNS enhances the expression of α7nAchR in the hypothalamus, activates the JAK2/STAT3 pathway, and inhibits the NF‐κB pathway, thus attenuating the secretion of IL‐1β and improving the depressive‐like behaviors in CUMS rats.

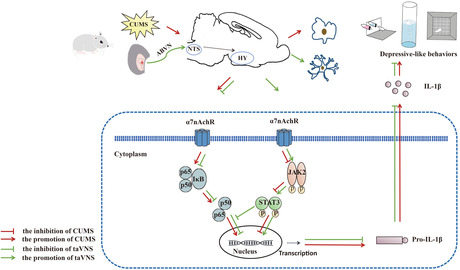

## 1 INTRODUCTION

Major depressive disorder (MDD) is a common recurrent psychiatric disorder with a high disability and suicide rate. *Diagnostic and Statistical Manual Of Mental Disorders* (Fifth edition) indicates that patients exhibit alterations in key functions such as mood, cognition, sleep, and appetite with a lack of pleasure as core symptom. According to the World Health Organization,^1^ depression affects more than 332 million people worldwide, and the high incidence of depression and suicide rates make it the number one cause of disability worldwide, causing not only suffering to the individual patient and their family but also a huge socioeconomic burden. Current therapies for depression are mainly pharmacotherapy, cognitive therapy, and physical therapy, with some adverse events during treatment, and the use of certain antidepressants can increase the risk of suicide in young people.^2^, ^3^

Inflammation is a prominent risk factor for depression. A prospective study showed that elevated biomarkers of inflammation preceded the onset of depressed mood in an aged population with no psychiatric history.^4^ The use of cyclooxygenase inhibitors in patients with MDD has also achieved promising results in the clinical.^5^ Peripheral inflammation can cause neuroinflammation by destroying the blood–brain barrier, which is the research focus in depression. In patients with MDD, increased cytokines and immune cells are found in the cerebrospinal fluid.^6^ The persistence of inflammation is also regarded as a major cause of depression relapse.^7^

Vagus nerve stimulation (VNS), one of the neuromodulation methods, has a favorable anti‐inflammatory effect and has been approved by the U.S. Food and Drug Administration as a complementary alternative therapy for refractory depression in 2005. Transcutaneous auricular vagus nerve stimulation (taVNS), which is developed from VNS, is a promising neuromodulation method for treating depression and could achieve the same effect as VNS without the traumatic damage caused by surgical implantation.^8^, ^9^ It works by stimulating the auricular branch of the vagus nerve (ABVN) in the cymba concha. ABVN has been proven to be the only body surface branch of the vagus nerve (VN) and transmits information to the nucleus of tract solitary (NTS), and then forms direct and indirect ascending projections from the NTS to the hypothalamus, amygdala, hippocampus, frontal lobes, and other areas of the brain,^10^, ^11^ which are thought to influence the pathogenesis of depression. A meta‐analysis showed that taVNS is effective in improving symptoms of MDD.^12^ Our previous studies also have demonstrated that taVNS improves default mode brain network connectivity in patients with MDD, with significantly better symptom relief and improvement than sham taVNS in depressed patients. Compared with sham taVNS, clinical improvements continued until the 12th week during taVNS.^13^, ^14^ Previous series of experiments also confirmed that taVNS suppresses hypothalamic inflammation and improves depression‐like behavior in animals, and it is thought to exert anti‐inflammatory effects through α7 nicotinic acetylcholine receptors (α7nAchR), for its inhibitory effect on TNF‐α can be partially eliminated by α7nAchR antagonists.^15^, ^16^ However, its exact molecular mechanism is not well clarified.

The α7nAchR is a subtype of nicotinic acetylcholine receptor, and an orchestrator of vagal inflammatory reflex, which refers to a physiological neuro‐immune mechanism that restricts inflammation.^17^ It has been shown that α7nAchR resident on macrophages can regulate inflammation by inhibiting cytokine release. The knockdown of α7nAchR and specific antagonist blockade both abrogate the anti‐inflammatory effects of the VN. The α7nAchR is one of the most abundantly expressed and widely distributed nicotinic acetylcholine receptors in the brain, expressed in the cortex, hypothalamus, and other brain regions.^18^ The hypothalamus can also influence emotion regulation.^19^ Currently, α7nAchR agonists are mainly investigated for neurodegenerative diseases and psychiatric disorders, mimicking stimulation of VN to produce immune activity.^20^, ^21^ Janus tyrosine kinase 2/signal transducer and activator of transcription 3 (JAK2/STAT3) and nuclear factor κB (NF‐κB) signal pathway, considered as a master regulator of inflammation, are thought to be associated with the anti‐inflammatory regulation of α7nAchR. The activation of NF‐κB and JAK2/STAT3 induces the expression of genes such as inflammatory cytokines and chemokines, amplifying or blocking the inflammatory cascade response. JAK2/STAT3 and NF‐κB signaling system may be new targets for depression treatment, and intervening in their activation pathway, it also has positive implications for the treatment of depression.^22^, ^23^

In this study, we explored whether taVNS could improve depression‐like behaviors and suppress neuroinflammation in chronic unpredicted mild stress (CUMS)‐exposed rats, and further explored the molecular mechanisms underlying the action of taVNS, whether its antidepressant effect based on anti‐inflammation is mediated by the regulation of α7nAchR via JAK2/STAT3/NF‐κB signaling pathway. Specifically, we examined behaviors, which include anhedonia and behavioral despair, and detected the level of α7nAchR and related neuroinflammatory factors in the hypothalamus, such as microglia, NF‐κB p65, IL‐1β, phosphorylated JAK2, phosphorylated STAT3, and so on, by using homozygous α7nAchR^−/−^ gene knockout rats.

## 2 METHOD AND ANIMALS

### 2.1 Experimental animals

Specific Pathogen Free healthy adult male Sprague–Dawley (SD) rats (200 ± 20 g) were obtained from the Laboratory Animal Center of Academy of Military Medical Sciences. Homozygous α7nAchR^−/−^ gene knockout (200 ± 20 g) was obtained from Saiye (Guangzhou) Biotech Co., LTD. The rats were housed in standard laboratory conditions under a 12‐h light‐dark circle (lights on at 7:00 a.m.), (55 ± 5) % humidity, and a temperature of 20–25°C with ad libitum access to food and water except when animals were subjected to stressors during the CUMS procedure. All animal experimental procedures were strictly performed according to the Guide for the Care and Use of Laboratory Animals of the Ministry of Science and Technology of the People's Republic of China, and the protocol was approved by the Ethics Committee of Institute of Acupuncture and Moxibustion, China Academy of Chinese Medical Sciences (Permit No. D2019‐02‐11‐3).

### 2.2 Experimental design

**FIGURE 1:**
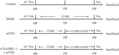
The Schematic representation of experimental protocol. AC, acclimatization; CUMS, chronic unpredictable mild stress; taVNS, transcutaneous auricular vagus nerve stimulation; test includes body weight measurement, sucrose preference test, forced swimming test, and open field test.

After 1 week of adaptive feeding, the rats were randomly allocated to control, model, taVNS, and α7nAchR^−/−^ + taVNS group (*n* = 10 each group). Except for the control group, other groups were subjected to single cage rearing and CUMS for 6 weeks. TaVNS and α7nAchR^−/−^ + taVNS group were administrated to taVNS for 3 weeks at the 4th week. At the beginning and the end of CUMS modeling, behavioral tests, and weight measurement were carried out, and the rats were sacrificed under anesthesia with an intraperitoneal injection of 1% sodium pentobarbital (50 mg/kg) and hypothalamus were collected. The whole experimental protocol is shown in Figure 1.

### 2.3 Chronic unpredicted mild stress procedure

The CUMS procedure was performed for induction of depression in a rat model as previously described^24^ with minor modifications. The following stressors were imposed for 6 consecutive weeks: tail pinch for 3 min, swimming in cold water (4°C) for 5 min, food deprivation for 24 h, sawdust bedding for 24 h, water deprivation for 24 h, electric shock feet (1 mA, 10 s each time, 10 s interval, 2 min), and alterations of light/dark cycle. All stressors were applied individually, continuously, and randomly to produce an unexpected mild stress effect. Control animals were left uninterrupted, except for regular cage cleaning.

### 2.4 Transcutaneous auricular vagus nerve stimulation

**FIGURE 2:**
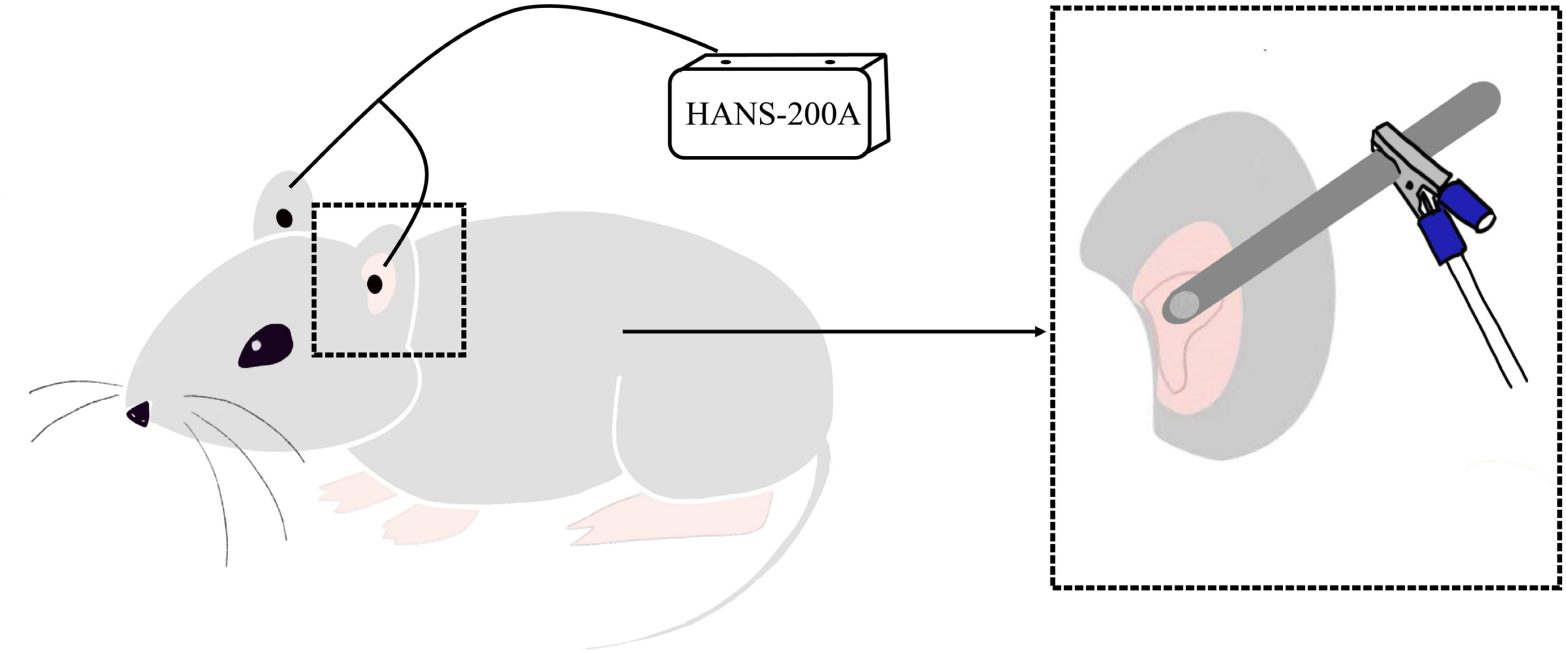
The diagram of transcutaneous auricular vagus nerve stimulation. The site of electrical stimulation is shown in the enlarged part of the above figure, which is the distribution area of the auricular branch of the vagus nerve.

The protocol of taVNS was performed based on a previous study. Mild anesthesia was performed with isoflurane (0.5%–1% oxygen) inhaled from the nasal cone using a desktop animal anesthesia ventilator system (VME, Matrix). Bilateral magnetic electrodes placed noninvasively at the auricular concha were connected via a handmade metal ear splint (2 cm in length, 0.5 cm in width, and 0.05 cm in thickness) to an electroacupuncture stimulator (HANS‐200A, Nanjing Jisheng Medical Technology Co., Ltd.) (see Figure 2). The stimulation parameters were as follows: 2 ms square pulses (2 mA) at 2/15 Hz (2 and 15 Hz, switched every second) were maintained for 30 min per day. Evaluate the conductive effect by slight vibrations in the auricle. Swab the auricle with a cotton swab dipped in normal saline to enhance the conductive effect without vibration. The intervention time started from 8:00 to 11:00 a.m. every day.

### 2.5 Behavioral tests

All the behavioral tests followed the sequence of sucrose preference test (SPT), open field test (OFT), and forced swimming test (FST) as previously described.^25^, ^26^ Behavioral tests were carried out in a quiet and undisturbed condition.

#### 2.5.1 Sucrose preference test

The SPT was performed as in our previous study. Rats were habituated to consume 1% sucrose solution and pure water from different bottles for 24 h before conducting the test. After fasting and water deprivation for the following 24 h, the rats were all housed in individual cages and had free access to two pre‐weighed bottles containing 1% sucrose solution and pure water for 2 h. To avoid place preference, the two bottles were switched after 1 h. At the end of the test, the bottles of 1% sucrose solution and pure water were re‐weighted and recorded. The sucrose preference ratio, an evaluation of anhedonia, which is one of the core symptoms of depressive disorder, was calculated as follows: sucrose preference ratio (%) = sucrose water consumption/(sucrose water consumption + pure water consumption) × 100%.

#### 2.5.2 Open field test

The apparatus was a black plastic box with 25 equal squares (100 cm in diameter with 40 cm boundary walls). Each rat was gently placed in the center of the square and allowed to explore freely for 5 min. The apparatus was cleaned with 75% ethanol to remove residual olfactory cues before each test. The locomotor activity of each animal was monitored for 5 min and analyzed by a video analysis system (Shanghai Jiliang Software Technology Co., Ltd.).

#### 2.5.3 Forced swimming test

The FST was performed as described previously with minor modifications. The rats were habituated to force to swim individually in an open cylinder container (30 cm in diameter and 46 cm in height) with water (24 ± 2°C) for 15 min on the day before the experiment. The rats were exposed to the same conditions in the formal experiment for 5 min. Total immobility time was recorded during the 3 min testing period. The immobility time, commonly used to reflect the level of desperation of the rodents, was defined as floating with only necessary small movements to keep the head above water.

### 2.6 Immunofluorescent staining

Rats were intracardially perfused with saline and 4% paraformaldehyde. The brain tissues were harvested and post‐fixed for 2 h and dehydrated in 25% sucrose solution for the day before sectioning (slice thickness: 30 μm). Free‐floating sections blocked with 5% normal donkey serum in 0.1 M PB and 0.5% Triton X‐100 (30 min, room temperature, RT). Then the primary antibody incubation was conducted (overnight, 4°C). After washing with 0.1 M PB for three times, fluorescent secondary antibodies were applied (2 h, RT). Sections were washed as mentioned above and mounted in a mounting medium containing DAPI. The antibodies used were as follows: anti‐ionized calcium‐binding adapter molecule 1 (Iba1) (goat polyclonal, 1:200, Abcam/ab5076), anti‐IL‐1β (rabbit polyclonal, 1:50, Abcam/ab9722), Alexa Fluor® 488‐conjugated donkey anti‐rabbit IgG (1:300, Abcam/ab150073), donkey anti‐goat IgG H&L Cy3® (1:300, Abcam/ab6949). Incubation was conducted at a shaker condition. The images were captured with the confocal microscope (Olympus).

### 2.7 Western blot analysis

The total protein extracted from the hypothalamus was homogenized in RIPA lysis buffer, and subsequently, the concentration of the supernatants was quantified by bicinchoninic acid assay. The proteins were separated by sodium dodecyl sulfate‐polyacrylamide gel electrophoresis and then transferred to polyvinylidene difluoride membranes, which were then blocked in TBST (Tris‐buffered saline, containing Tween‐20) with 5% skim milk (1 h, RT). The membranes were incubated with the following primary antibodies at 4°C with shaking overnight: NF‐κB p65 (1:1000, rabbit monoclonal, CST/8242); Phospho‐NF‐κB p65 (1:1000, rabbit monoclonal, CST/3033); IL‐1β (1:500, rabbit polyclonal, Abcam/ab9722); α7nAchR (1:500, rabbit polyclonal, Abcam/ab10096); Phospho‐JAK2 (1:1000, rabbit monoclonal, CST/3776); Phospho‐STAT3 (1:2000, rabbit monoclonal, Abcam/ab76315); β‐actin (1:3000, mouse monoclonal, Abcam/ab6276). After washing, the membranes were further incubated (1 h, RT) with anti‐mouse and anti‐rabbit secondary horse radish peroxidase‐conjugated antibodies (1:5000, respectively; goat anti‐mouse, Abcam/ab6789; goat anti‐rabbit, Abcam/ab6721). The immunoblotting bands were detected via an Image Quant LAS4000 mini image analyzer (GE Healthcare), and then quantified with Quantity One software v.4.6.2 (Bio‐Rad).

### 2.8 Data analysis

The Statistical Package for the Social Science (SPSS) version 26.0 (IBM, Armonk) was used for the statistical analyses. Shapiro–Wilk test was used to evaluate the normality of the data distribution. Parameter tests were performed by one‐way analysis of variance test (ANOVA) followed by Tukey's post hoc multiple comparisons. Non‐parameter tests were performed by Kruskal–Wallis (K‐W). All analyses were performed using Graphpad Prism 8.0 (Graphpad Software Inc.). The data were presented as mean ± standard deviation, and *p* < 0.05 was considered statistically significant.

## 3 RESULTS

### 3.1 Effect of transcutaneous auricular vagus nerve stimulation on body weight and depression‐like behaviors in chronic unpredicted mild stress–exposed rats and chronic unpredicted mild stress–exposed α7nAchR ^−/−^ rats

**FIGURE 3:**
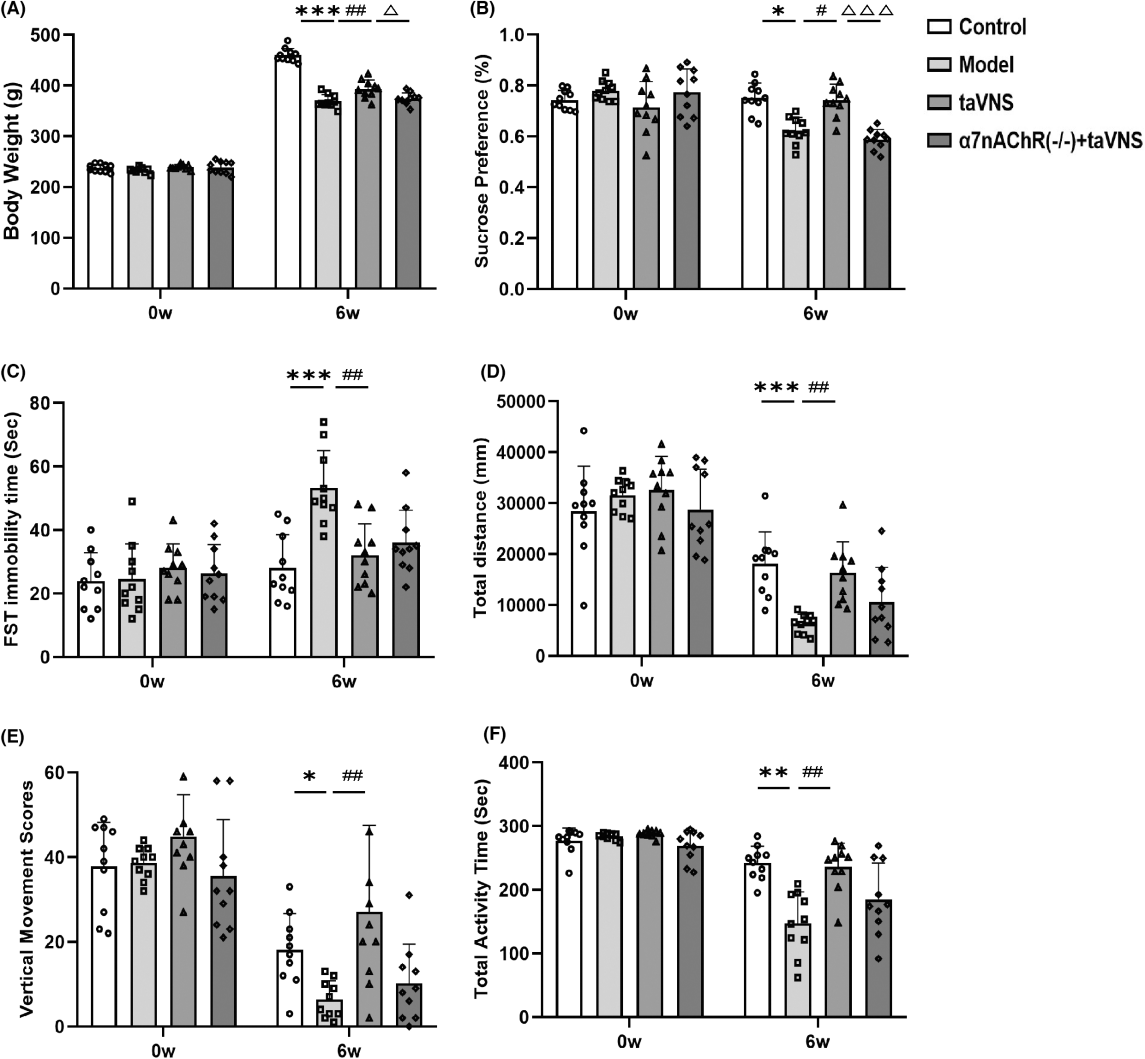
The effect of transcutaneous auricular vagus nerve stimulation (taVNS) on the body weight and behavioral tests of rats at different time points. (A) Comparison of body weight of rats at different time points. (B) Comparison of sucrose preference of rats at different time points. (C) Comparison of immobility time of rats in FST at different time points. (D) Comparison of the total distance of rats in open field test (OFT) at different time points. (E) Comparison of the vertical movement scores of rats in OFT at different time points. (F) Comparison of the total activity time of rats in OFT at different time points. *p < 0.05 versus the control group, **p < 0.01 versus the control group, ***p < 0.001 versus the control group; ^#^ *p* < 0.05 versus the model group, ^##^ *p* < 0.01 versus the model group; ^△^ *p* < 0.05 versus the taVNS group, ^△△△^ *p* < 0.001 versus the taVNS group (*n* = 10 for each group).

To investigate the effects of taVNS on CUMS rats, rats were evaluated by body weight measurement and several behavioral tests including SPT, FST, and OFT as successively shown in Figure 3.

#### 3.1.1 Body weight

No statistical difference at 0 week of body weight (Figure 3A, *p* > 0.05). At 6 week, compared with the control group, the model group exhibited a remarkable weight loss (Figure 3A, *p* < 0.001, vs. Control). TaVNS has an evident increasing impact on the body weight of CUMS‐exposed rats (Figure 3A, *p* < 0.01, vs. Model). However, CUMS‐exposed α7nAchR^−/−^ rats showed a decreasing body weight compared with the taVNS group (Figure 3A, *p* < 0.05, vs. taVNS).

#### 3.1.2 SPT

At 0 week, there was no significant difference in sucrose preference among groups (Figure 3B, *p* > 0.05). At 6 week, the sucrose preference ratio significantly decreased after exposure to chronic stress (Figure 3B, *p* < 0.05, vs. Control). TaVNS rescued the reduction of sucrose preference ratio (Figure 3B, *p* < 0.05, vs. Model), but compared with the taVNS group, the sucrose preference ratio of rats in α7nAchR^−/−^ + taVNS group decreased (Figure 3B, *p* < 0.001, vs. taVNS).

#### 3.1.3 Forced swimming test

There was no significant difference in immobility time among groups at 0 week (Figure 3C, *p* > 0.05). At 6 week, an increasing FST immobility time was observed in the model group compared with the control group (Figure 3C, *p* < 0.001, vs. Control). TaVNS decreased the immobility time in the CUMS‐exposed rats (Figure 3C, *p* < 0.01, vs. Model). But it has shown an upward trend in immobility time in CUMS‐exposed α7nAchR^−/−^ rats.

#### 3.1.4 Open field test

There was no significant difference in OFT results among groups at 0 week (Figure 3D–F, *p* > 0.05). At 6 weeks, the model group exhibited a series of depression‐like behaviors, including a decreasing total distance of spontaneous exploration movement, vertical movement score, and activity (Figure 3D–F, *p* < 0.001, *p* < 0.05, *p* < 0.01, respectively, vs. Control). These depressive symptoms can be reversed by taVNS, in a form of an obviously increasing total distance of spontaneous exploration movement, vertical movement score, and activity time (Figure 3D–F, *p* < 0.01, *p* < 0.01, *p* < 0.01, respectively, vs. Model) compared with the model group. The improving effect of taVNS on spontaneous exploration movement, vertical movement score, and activity time in OFT weakened in α7nAchR^−/−^ + taVNS group compared with the taVNS group, with a downward trend in total distance of spontaneous exploration movement, vertical movement score, and activity time.

### 3.2 The effect of transcutaneous auricular vagus nerve stimulation on hypothalamic microglia and IL‐1β

**FIGURE 4:**
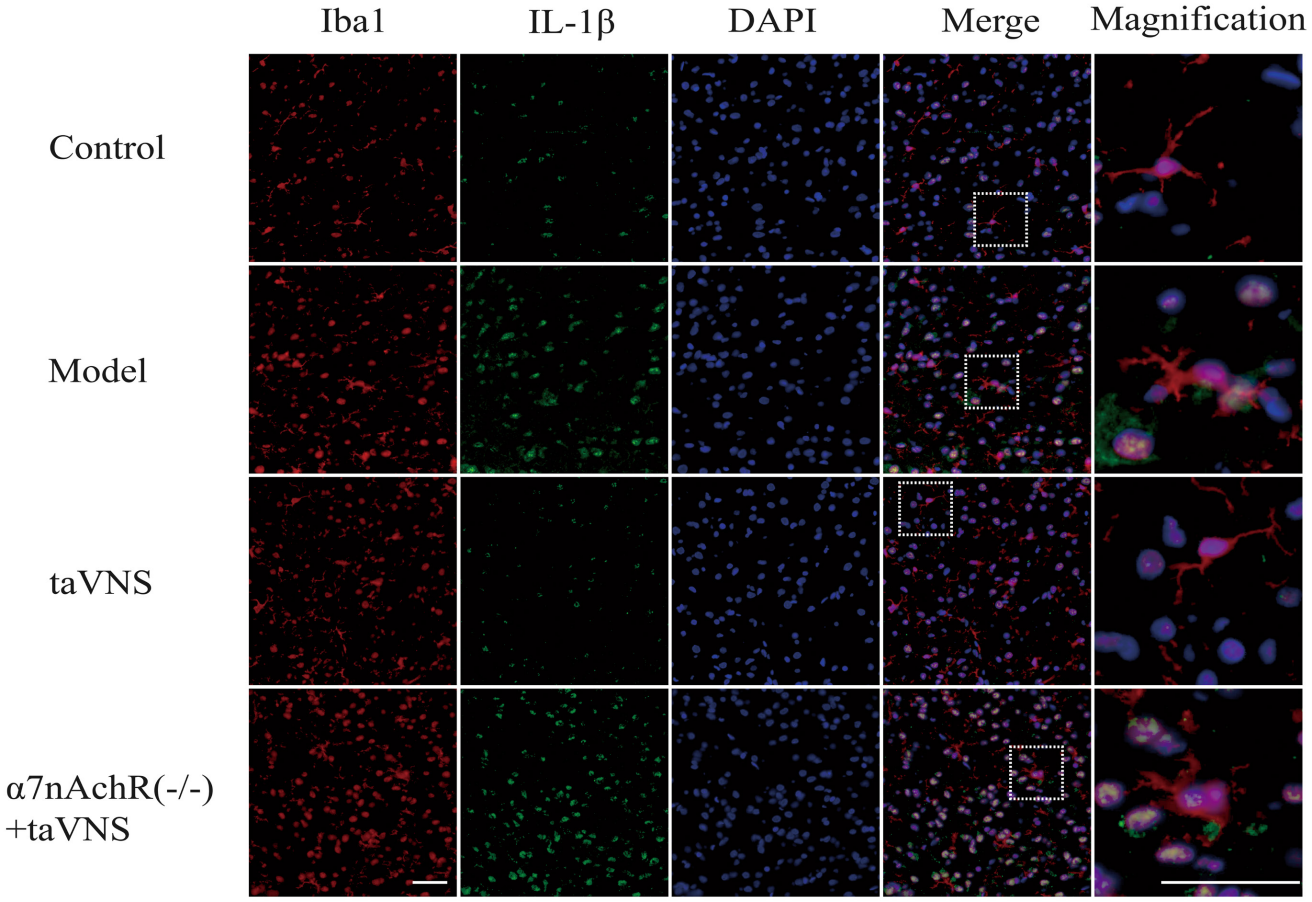
Immunofluorescence staining for microglia (Iba1) and IL‐1β in the hypothalamus. The morphology of microglia was transformed by taVNS from ameboid state to resting state in CUMS‐exposed rats in the hypothalamus. The microglia in α7nAchR^−/−^ +  taVNS group were in the activated state. The expression of IL‐1β was increased in the model group compared with the control group, and taVNS decreased the expression compared with the model group. The expression in α7nAchR^−/−^ + taVNS group was increased compared with the taVNS group. The microglia marker (Iba1) is in red, IL‐1β is in green, and the nuclear marker (DAPI) is in blue, scale bar: 50 um.

As shown in Figure 4, the morphology of microglia in the hypothalamus was shown by immunofluorescent. Microglia were activated by CUMS exposure. Microglia in the control group were in a resting state, with comparatively small cell bodies and typical long, ramified, much elaborated thin processes, which send multiple branches and extend in all directions. Compared with the control group, microglia were activated that showed less ramified and amoeboid cell shape, characterized by swollen, truncated processes and enlarged cell bodies in the model group. Compared with the model group, microglia in the taVNS group were not obviously activated, but in a relatively resting state. The morphology of activated amoebic‐like microglia was observed in the α7nAchR^−/−^ + taVNS group. The results also revealed that pro‐inflammatory cytokine IL‐1β rarely expressed in the control group. The expression of IL‐1β was evidently increased in the model group compared with the control group, while in the taVNS group, the result was reversed. The expression of IL‐1β in α7nAchR^−/−^ + taVNS group increased compared with the taVNS group.

### 3.3 Effect of transcutaneous auricular vagus nerve stimulation on the activation of hypothalamic α7nAchR/JAK2/STAT3/NF‐κB in chronic unpredicted mild stress–exposed rats

**FIGURE 5:**
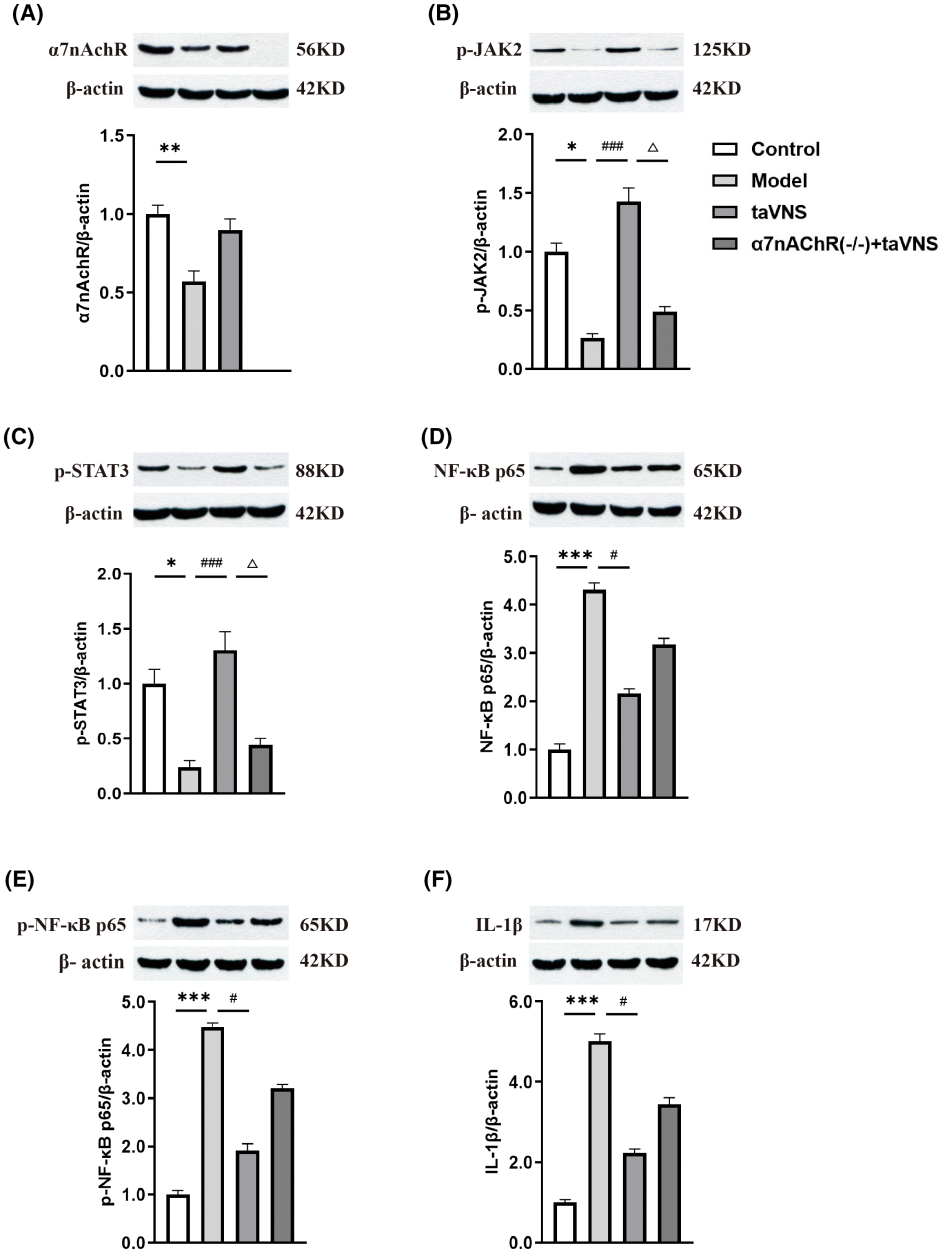
The effect of transcutaneous auricular vagus nerve stimulation (taVNS) on the expression of α7nAchR, p‐JAK2, p‐STAT3, NF‐kB p65, p‐NF‐kB p65, and IL‐1β in the hypothalamus. (A) Effect of taVNS on the expression of α7AchR in the hypothalamus of rats. (B) Effect of taVNS on the expression of p‐JAK2 in the hypothalamus of rats. (C) Effect of taVNS on the expression of p‐STAT3 in the hypothalamus of rats. (D) Effect of taVNS on the expression of NF‐kB p65 in the hypothalamus of rats. (E) Effect of taVNS on the expression of p‐NF‐kB p65 in the hypothalamus of rats. (F) Effect of taVNS on the expression of IL‐1β in the hypothalamus of rats. The values are presented as mean ± SD. *p < 0.05 versus the control group, **p < 0.01 versus the control group, ***p < 0.001 versus the control group; ^#^ *p* < 0.05 versus the model group, ^###^ *p* < 0.001 versus the model group; ^△^ *p* <0.05 versus the taVNS group (*n* = 6 for each group).

To explore potential molecular mechanisms underlying the antidepressant of taVNS, the expression of α7nAchR, phosphorylated‐JAK2 (p‐JAK2), phosphorylated‐STAT3 (p‐STAT3), IL‐1β, NF‐κB p65, and phosphorylated‐NF‐κB p65 (p‐NF‐κB p65) were examined by western blot as successively shown in Figure 5. The α7nAchR protein levels in the hypothalamus significantly decreased in the model group compared with the rats in the control group (Figure 5A, *p* < 0.01, vs. Control), and the levels of α7nAchR showed an upward trend after being subjected to 3 weeks of taVNS. The α7nAchR protein expression was not detectable in the α7nAchR^−/−^ rats. Compared with the control group, the protein expression of p‐JAK2 and p‐STAT3 decreased in the model group (Figure 5B,C, *p* < 0.05, *p* < 0.05, respectively, vs. Control). The IL‐1β, NF‐κB p65, and p‐NF‐κB p65 levels in the hypothalamus of CUMS‐exposed rats were increased significantly (Figure 5D–F, *p* < 0.001, *p* < 0.001, *p* < 0.001, respectively, vs. Control). The changes were reversed by the taVNS group. TaVNS not only rescued protein levels of p‐JAK2 and P‐STAT3 in the hypothalamus of CUMS rats, compared with the model group (Figure 5B,C, *p* < 0.001, *p* < 0.001, respectively, vs. Model) but also decreased the levels of IL‐1β, NF‐κB p65 and p‐NF‐κB p65 (Figure 5D–F, *p* < 0.05, *p* < 0.05, *p* < 0.05, respectively, vs. Model). The effect of taVNS was attenuated in α7nAchR^−/−^ + taVNS group, with decreasing expression of p‐JAK2 and p‐STAT3 (Figure 5B,C, *p* < 0.05, *p* < 0.05, respectively, vs. taVNS) and a upward trend expression of IL‐1β, NF‐κB p65 and p‐NF‐κB p65 (Figure 5D–F) compared with the taVNS group.

## 4 DISCUSSION

Major depressive disorder is a mental disease with complex pathogenesis, and neuroinflammation plays an important role in the development of depression in recent years. A meta‐analysis shows that antidepressants also play a role in reducing inflammatory factors.^27^ Anti‐inflammatory agents, used as new target drugs in the clinical treatment of depression, exert antidepressant effects in patients with MDD and have a reasonable safety profile.^28^ Based on the relationship between VN and neuroinflammation, this study innovatively explores the mechanism of taVNS from a hypothalamic cholinergic anti‐inflammatory perspective, revealing the molecular mechanism of taVNS antidepressant and providing better guidance for its clinical application.

Transcutaneous auricular vagus nerve stimulation exhibits the antidepressant‐like effect through α7nAchR in CUMS‐exposed rats. CUMS, performed by prolonged exposure to stressors to establish depression models, is one of the most widely used models of depression with high validity. Model evaluation methods for CUMS include weight measurement, SPT, FST, and OFT, which result in depression‐like symptoms and behaviors such as decreased body weight gain, decreased sucrose preference ratio, increased FST immobility time, and decreased OFT total distance of spontaneous exploration movement, vertical movement score and activity time.^29^ In the present study, the rats showed the above‐mentioned body weight changes and depression‐like behaviors after CUMS exposure, and these could be reversed by taVNS, indicating that taVNS could improve depression‐like behaviors and body weight in CUMS‐exposed rats. This is consistent with our previous findings.^30^, ^31^ And the antidepressant‐like effect of taVNS was attenuated in α7nAchR^−/−^ CUMS‐exposed rats. It has also been experimentally found that α7nAchR‐positive allosteric modulator can effectively prevent lipopolysaccharide‐induced anxiety, cognitive deficits, and depression‐like behaviors, and cholinergic stimulation or α7nAchR agonist injection can alleviate depression‐like behaviors in chronic restraint stress mice,^32^ and all of these can be prevented by α7nAchR antagonists. Thus, we speculate that α7nAchR may partially involve the antidepressant effects of taVNS.

Transcutaneous auricular vagus nerve stimulation has an anti‐inflammatory effect through VN immune activities. Clinical trials showed that taVNS attenuates the release of pro‐inflammatory factors in serum.^33^ TaVNS exerts anti‐inflammatory effects that have also been proven to be related to α7nAchR. The anti‐neuroinflammatory mechanism of electroacupuncture and taVNS may be related to the enhanced expression of α7nAchR protein by α7nAchR/JAK2/STAT3 and cholinergic factors in brain regions.^34^, ^35^, ^36^ Enhanced vagal signaling by external stimulation with upregulating α7nAchR expression can have a beneficial effect in the regulation of inflammatory factors and contribute to depression, a neuroinflammatory disease. One study found that taVNS reversed hippocampal neuroinflammatory responses by α7nAchR.^26^ We can conclude that α7nAchR is a key mediator contributing to vagal neuroinflammation. As a structure of the limbic system and a controller in the autonomic nervous system, with projective connections arising from the hippocampus, amygdala, and cortex, the hypothalamus is also connected with various emotional responses.^37^, ^38^ But the role of the hypothalamus in emotions has been rarely explained. The hypothalamus demonstrates a role in symptoms of depression, such as lack of rewarding feelings, eating, and disturbed cognitive functions.^39^ Targeting specific receptors in the hypothalamus is considered an emerging treatment for psychiatric disorders.^40^ Hypothalamic inflammation is also thought to be involved in the onset of depression. Depression causes activation of hypothalamic microglia and activation of the hypothalamic–pituitary–adrenal (HPA) axis. By suppressing hypothalamic inflammation, depression‐like behaviors can be restored in model animals.^41^, ^42^, ^43^ Based on the important role of the hypothalamus in depression and connection to the hippocampus in the limbic system, it is also a key projection brain area for the central effects of stimulation of ABVN, in the present study, we focused on changes in α7nAchR in the hypothalamus.

Abnormal expression of α7nAchR causes behavioral abnormalities in rodents,^44^ suggesting that α7nAchR may be a new therapeutic target for the prevention and treatment of depression. And some nicotinic compounds used in psychiatric disorders are thought to act by α7nAchR, and α7nAchR agonists or antagonists are also used in the treatment of depression.^45^, ^46^ A series of studies confirmed that activation of α7nAchR in microglia induces anti‐inflammatory effects.^47^, ^48^ The α7nAchR on microglia plays an essential role in depression. Cholinergic stimulation or α7nAchR agonists alleviate neuroinflammation induced by acute or chronic depression models and reduce positive expression of Iba1, which can be blocked by selective α7nAchR antagonists. In chronic depression models, the activation of α7nAchR also restores central cholinergic signaling.^32^, ^49^ The α7nAchR are widely expressed in the hypothalamus, one of the structures that undergoes the most pronounced alterations in depression. Our results showed that taVNS inhibited the activation of hypothalamic microglia and reduced the expression of IL‐1β and transformed the activated microglia into a resting state in CUMS‐exposed rats. However, this effect was attenuated in α7nAchR^−/−^ rats.

**FIGURE 6:**
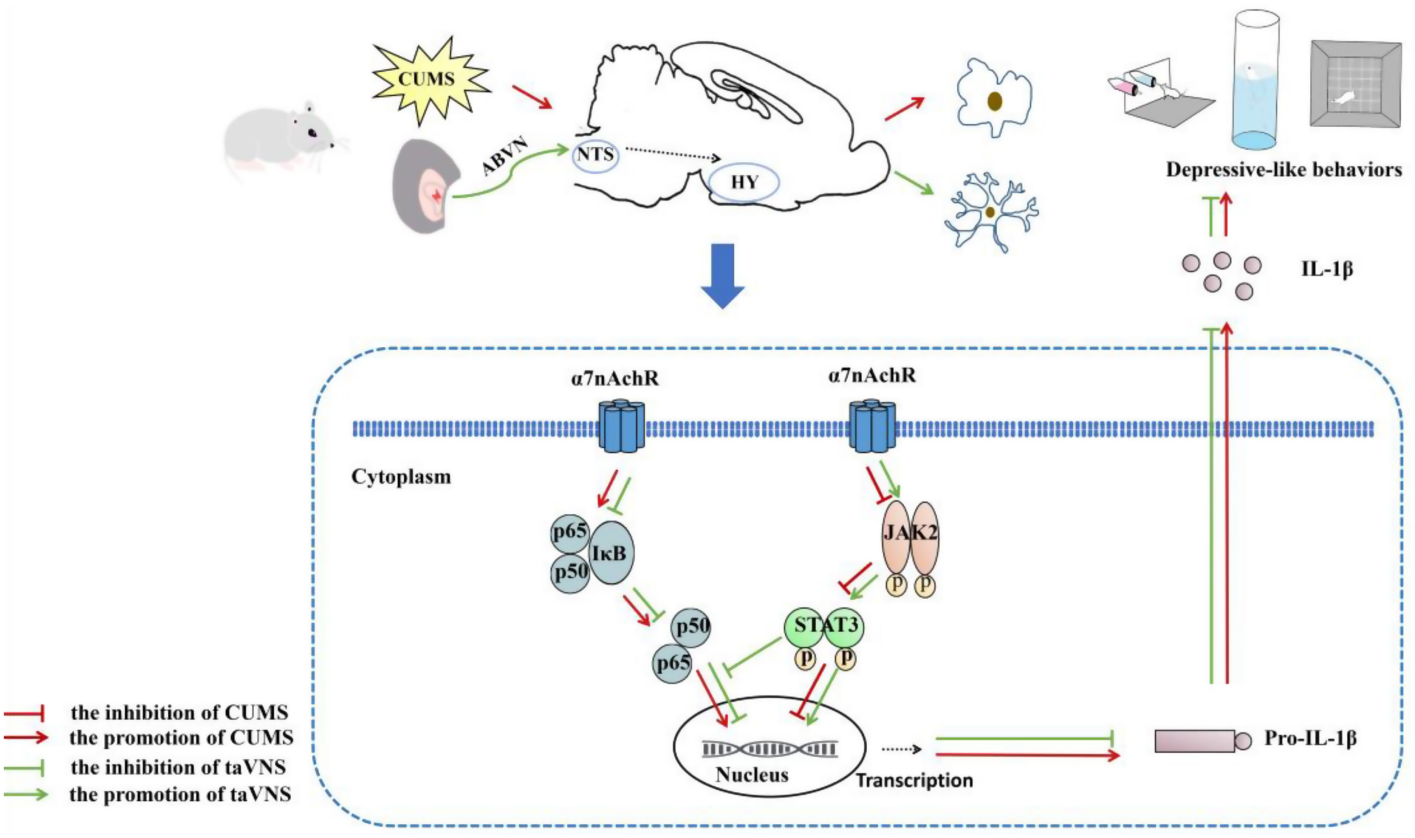
Anti‐neuroinflammation effects of taVNS against depressive‐like behaviors may be related to the regulation of hypothalamic α7nAchR/JAK2/STAT3/NF‐κB signal pathway. taVNS stimulates ABVN, and the information from the vagus afferent fibers project to the NTS and then form ascending projections to the hypothalamus, which inhibits the activated microglia. taVNS enhances the expression of α7nAchR, to phosphorylate JAK2 to p‐JAK2, then phosphorylate STAT3 to p‐STAT3, and inhibit the phosphorylation of NF‐κB p65 signal pathway, thus attenuating the secretion of IL‐1β and improve depressive‐like behaviors in CUMS‐exposed rats. ABVN, the auricular branch of the vagus nerve; CUMS, chronic unpredictable mild stress; HY, hypothalamus; NTS, nucleus of tract solitary; taVNS, transcutaneous auricular vagus nerve stimulation; α7nAchR, α7 nicotinic acetylcholine receptor.

Activation of the α7nAchR activates a series of anti‐inflammatory cascades that regulate multiple intracellular signaling cascades in monocytes and macrophages. The activation of α7nAchR can phosphorylate and activate JAK2, which then recruits and phosphorylates the transcription factor STAT3, promoting the release of the anti‐inflammatory factors. And the activation of α7nAchR also can inhibit phosphorylation of IκB and further prevent activation of the NF‐κB pathway, thereby inhibiting the transcription of pro‐inflammatory cytokines. JAK2/STAT3 is thought to be a key bridge between α7nAchR and NF‐κB regulation. The phosphorylation of STAT3 can mimic α7nAchR signaling and inhibit the activation of NF‐κB.^41^ The JAK2/STAT3 and NF‐κB pathway have been well described in depression. JAK2/STAT3 pathway mediates the efficacy of antidepressants, and inhibition of the NF‐κB pathway suppresses the inflammatory response and restores depressive behavior in mice.^50^, ^51^ In our study, it showed that exposure to chronic stress for 6 weeks increased the release of the pro‐inflammatory factor IL‐1β by inhibiting α7nAchR in the hypothalamus of CUMS‐exposed rats, suppressing JAK2/STAT3 and activating the NF‐κB signaling pathway. In contrast, taVNS increased the expression of α7nAchR in the hypothalamus of model rats and reversed the above‐mentioned alterations in the hypothalamic signaling pathway. This suggests that taVNS may regulate the JAK2/STAT3 and NF‐κB signaling pathways by modulating the expression of α7nAchR, a finding further validated in CUMS‐exposed α7nAchR^−/−^ rats subjected to taVNS intervention, with decreased expression of p‐JAK2, p‐STAT3, increased expression of NF‐κB P65, p‐NF‐κB p65, and IL‐1β (see Figure 6).

The activation of α7nAchR in the hypothalamus may inhibit inflammation and restores the hypothalamic inflammation that causes weight changes in obesity, diabetes, and other disorders such as depression.^52^, ^53^, ^54^ Our previous experiments have demonstrated the effectiveness of taVNS in treating diabetic‐depression comorbidity.^55^ So taVNS probably is a new treatment for depression co‐morbidities, and α7nAchR in the hypothalamus may be one of the key mechanisms. Central inflammation is a crucial pathological mechanism in many neurodegenerative diseases such as Alzheimer's disease and Parkinson's disease, both of them are also diseases associated with the cholinergic system.^56^, ^57^ TaVNS can effectively improve central inflammation, and it may be a promising treatment for neurodegenerative diseases.

## 5 LIMITATIONS

This study has some obvious limitations. To avoid the interference of female sex steroid hormones, we used only male rats and did not address sex‐related differences, which are limited to the generalizability of our results. Because sexual dimorphism occurs in brain structure and gene expression, neuroinflammatory response, cerebral blood flow and metabolism, and stress‐induced psychiatric disorders in rats,^58^, ^59^, ^60^, ^61^, ^62^ we plan to investigate sex‐related differences in ameliorating taVNS on depression‐like behaviors in future studies.

## 6 CONCLUSION

In conclusion, taVNS ameliorates depression‐like behaviors in CUMS model rats, inhibits the hypothalamic microglia activity and increases α7nAchR expression in the hypothalamus, which in turn activates the JAK2/STAT3 signaling pathway and inhibits the NF‐κB signaling pathway. While its above effects are attenuated in α7nAchR^−/−^ rats. Available experimental results support the hypothesis that the hypothalamic α7nAchR/JAK2/STAT3/NF‐κB signaling pathway is one of the mechanisms by which taVNS exerts anti‐inflammatory to antidepressant effects.

## AUTHOR CONTRIBUTIONS

YC and YZ performed the experiments, and YC wrote the manuscript. JYW designed the experiments. YZ, JYW, YW, SYL, and XC reviewed the article. YFW, ZXZ, and JLZ provided experimental help. YW and PJR planned the overall direction of the experiment, supervised the experiment, and reviewed the article.

## FUNDING INFORMATION

This study was supported by the National Key R&D Program of China (2022YFC3500, 2022YFC3500501, 2018YFC1705800, 2018YFC1705803) the Science and Technology Innovation Project of China Academy of Chinese Medical Sciences (CI2021A03405).

## CONFLICT OF INTEREST STATEMENT

All authors declare that they have no conflict of interest.

## Data Availability

The data sets used in the current study are applicable from the corresponding author on reasonable requests.
